# A case report of non-functional ectopic left kidney obstructing the right kidney in crossed fused kidneys: A rare entity

**DOI:** 10.1016/j.ijscr.2021.106321

**Published:** 2021-08-19

**Authors:** Amine Hermi, Mokhtar Bibi, Kheireddine Mrad Dali, Houssem Hadj Alouane, Sami Ben Rhouma, Yassine Nouira

**Affiliations:** University Tunis Manar, Faculty of Medicine of Tunis, Department of Urology, La Rabta Hospital, Tunis, Tunisia

**Keywords:** Crossed fused renal ectopia, Renal ectopia, Nephrectomy, Pyelonephritis, Case report

## Abstract

**Introduction and importance:**

Crossed fused renal ectopia is a rare congenital condition that might pose some diagnostic and therapeutic challenges to clinicians. We report a patient with a non-functional crossed fused ectopic left kidney that obstructed the orthotopic kidney in a rarely observed situation.

**Case presentation:**

A 68-year-old male presented a right flank pain with fever. The diagnosis of right obstructive pyelonephritis was dressed, after biological and radiological investigations. The obstacle was a crossed ectopic left kidney in its inferior variety. The ectopic kidney was non-functional as result of an obstructive ureteral calculus. The patient had right ureteral stenting with a double-J catheter. Three months later, left nephrectomy was performed by lumbotomy. Per operative difficulties were mainly the infiltration of peri renal fat, the anarchic vascularization and the multiple small pedicles of the ectopic kidney that was also malrotated with the hilum facing anteriorly. Postoperative recovery was uneventful and the patient left the hospital after three days.

**Clinical discussion:**

Crossed Fused renal ectopy is rare. As shown in this case, the ectopic kidney might cause damage to the orthotopic kidney, by compression to urinary ducts. Surgery is the main treatment option. Some difficulties related to aberrant vascularization and possible malrotation is to preview.

**Conclusion:**

Crossed fused renal ectopia is uncommon renal anomaly, mostly asymptomatic. However, it may be responsible of some complications, sometimes severe. Surgery can be delicate due to vascular complexity.

## Highlights

• Crossed renal fused ectopy is rare and asymptomatic congenital condition. Association to other malformations is possible.
• The ectopic kidney may be responsible of the obstruction and the mal function of the orthotopic kidney.
• This is the first case to be reported in literature.
• Surgery might be difficult due to aberrant vascularization and possible malrotation of the kidney.

## 1 Introduction

Crossed fused renal ectopia (CFRE) is a rare congenital ectopia of one kidney to the controlateral side with fusion to the kidney on the same side. It counts approximately 1/7500 autopsy and live-births [1]. Renal vascularisation is variable with unclassic distribution. Surgery on kidneys with such abnormality might be delicate and risky. We herein report a case of partial nephrectomy on crossed fused kidneys, where the ectopic kidney was obstructive to the orthotopic kidney. This work is reported in line with the SCARE 2020 criteria [2].

## 2 Presentation of case

A 68-year-old male patient presented with right lumbo-abdominal pain with fever evolving since three days. Clinical examination showed a right flank tenderness, with no lumbar contact. He had no significant medical or psychological history, nor drug administration.

Laboratory investigations revealed hyperleukocytosis and high level of C-reactive protein. Creatinine clearance was at normal rate.

**Fig. 1:**
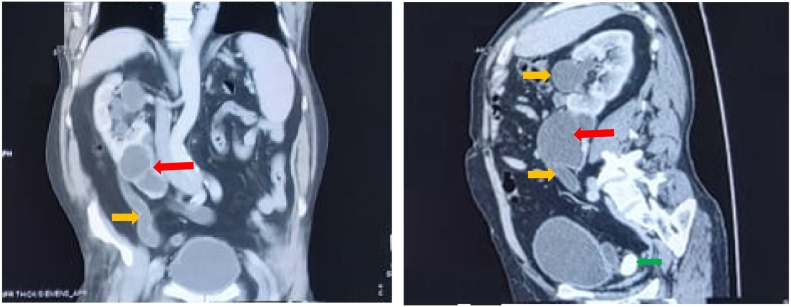
Frontal and sagittal views of CT showing crossed ectopic fused left kidney. The ectopic left kidney (red arrow ), with laminated parenchyma, is fused to the inferior pole of the right kidney, and was responsible of the obstruction and distension of the right ureter and pelvicalyceal (yellow arrow ). Image of ureteral calculi is shown by green arrow .

An abdominal computed tomography with contrast medium and delayed phases was performed and showed the absence of the left kidney in the left renal fossa. An ectopic kidney was identified on the right side fused to the lower pole of a right bulky kidney that was visualized in its normal position. The left kidney also appeared to be malrotated, with hilum facing anteriorly. The ureter of the left kidney crossed the midline at the level of lower third of the S1 vertebral body, and entered the bladder on the left side. The ectopic small kidney was non-functioning with an image of a left pelvic ureteral stone of 29 mm. Right hydronephrosis was found, with infiltration of peri renal fat signing the diagnosis of obstructive pyelonephritis, caused the ectopic kidney repressing the right ureter in its lumber portion. No associated congenital skeletal or Genito-urinary abnormalities were found (Fig. 1).

**Fig. 2:**
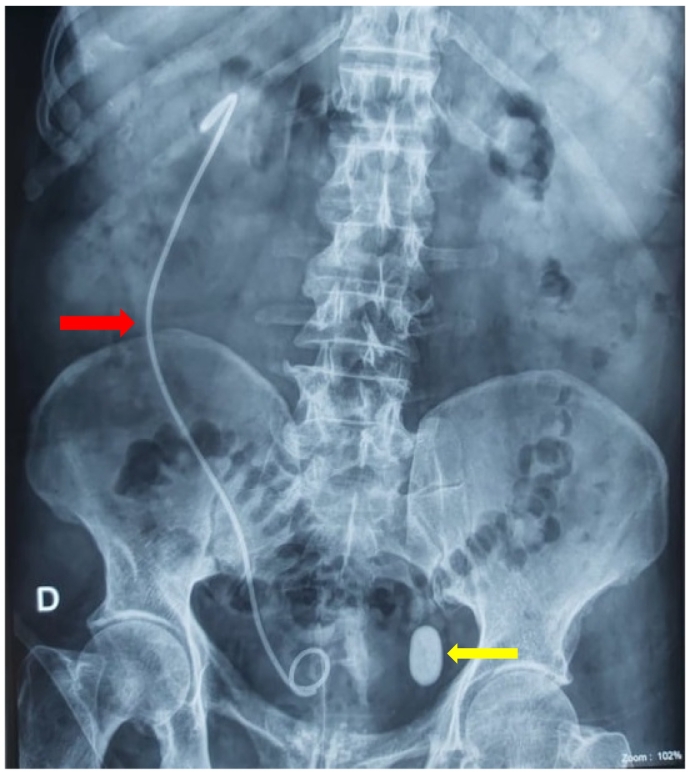
KUB showing a right double J ureteral stent with a 30 mm opacity (yellow arrow ) projected on the left half of bladder area. The double J stent (red arrow ) appears deviated to the right signing the deviation of the right ureter by the ectopic left kidney.

Antibiotic therapy was initiated and the patient had right ureteral stenting with a double-J catheter (Fig. 2). Postoperative recovery was uneventful, and the patient was discharged after 2 days on oral antibiotics and analgesics.

**Fig. 3:**
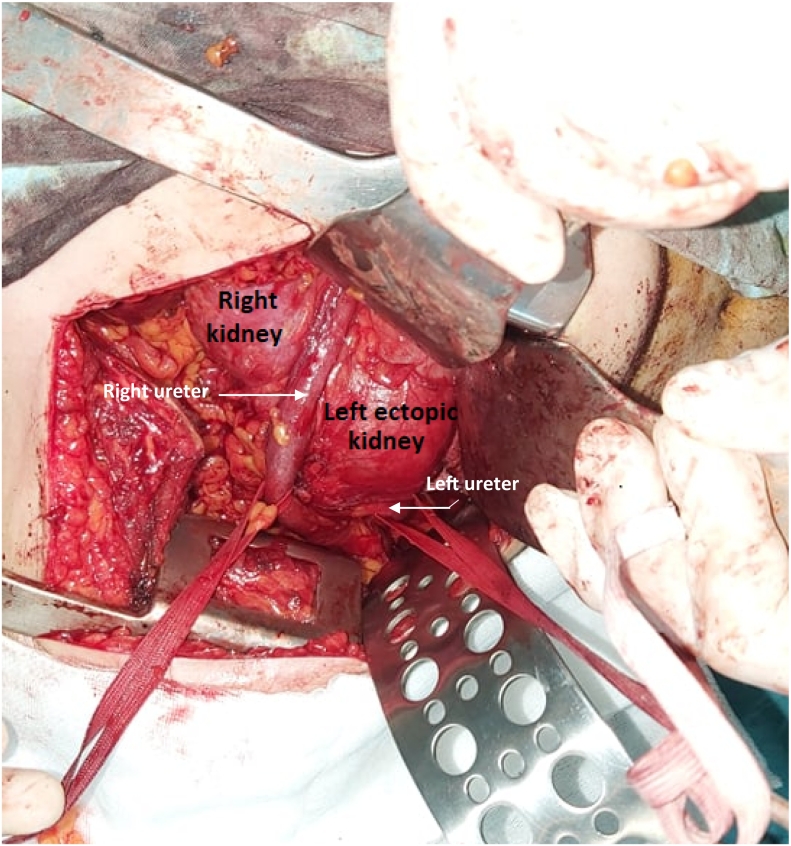
The two ureters, crossing each other, put on vascular loop. The right ureter crossing over the ectopic left kidney delineating the fusion line between both kidneys.

Three months later, nephrectomy of the non-functioning ectopic kidney was performed by lumbotomy, by experienced hands. After a retroperitoneal dissection, both ureters were identified and put on vascular loop. The right ureter was dilated and crossed over the ectopic left kidney pelvis deliniating the fusion line between both kidneys (Fig. 3). The left ureter was followed to its near insertion in the pelvis and dissected. The left crossed ectopic kidney was in antero superior rotation, and appeared fused at its upper pole to the lower pole of the right kidney. The dissection of both kidneys was difficult due to the infiltration of the peri renal fat.

The vascularization of the left ectopic kidney was anarchic and multiples small pedicles were found, penetrating inside and outside the renal sinus. Aberrant veins and arteries originating from the primitive and internal iliac vessels, draining the left ectopic kidney were identified. Careful ligature and section of these pedicles were performed and heminephrectomy was done. No urinary tract leakage was observed. The cortical edges of the resection margin were approximated in 2 plans with running resorbable suture using Vicryl 2-0 and 1 (Ethicon). After confirming complete hemostasis, a drainage tube was placed in the lumbar fossa, and the incision was closed by interrupted sutures. The postoperative course was uneventful. The patient was discharged on the third postoperative day. Histological examination of the operative specimens showed chronic pyelonephritis. A cytobacteriological examination of the urine was performed one month postoperative and showed no bacteriuria. No symptoms were reported. The renal function test was normal. A radiological control is programmed to be done in three months.

## 3 Discussion

Congenital abnormalities of the kidneys represent about 30% of all prenatally diagnosed malformations [2]. Second in incidence (approximately 1/7500) [3], [4] after horse-shoe kidney in congenital renal fusion abnormality, CFRE is three times more common to have a left-to-right crossover. It was most commonly reported in men [5].

In this entity, one of the kidneys crosses the midline and comes to lie on the opposite side and is fused to the inferior pole of its mate. The ureter of the ectopic kidney crosses the midline, emptying into the bladder on the opposite side [6]. The resulting renal mass varies in anatomic form according to the portion of the kidneys involved in the fusion.

Crossed renal ectopia may be divided into four types (Table 1). Six different forms of CFRE have been described [4] (Table 2).

**Table 1:**
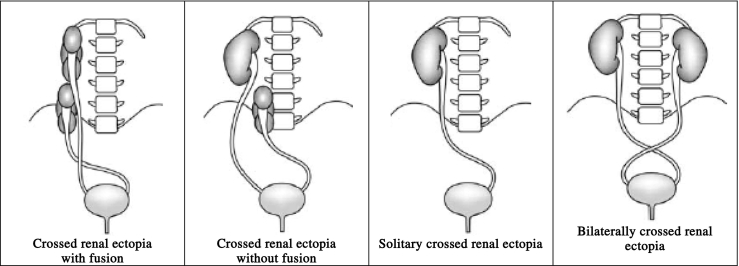
The four types of crossed renal ectopia.

**Table 2:**
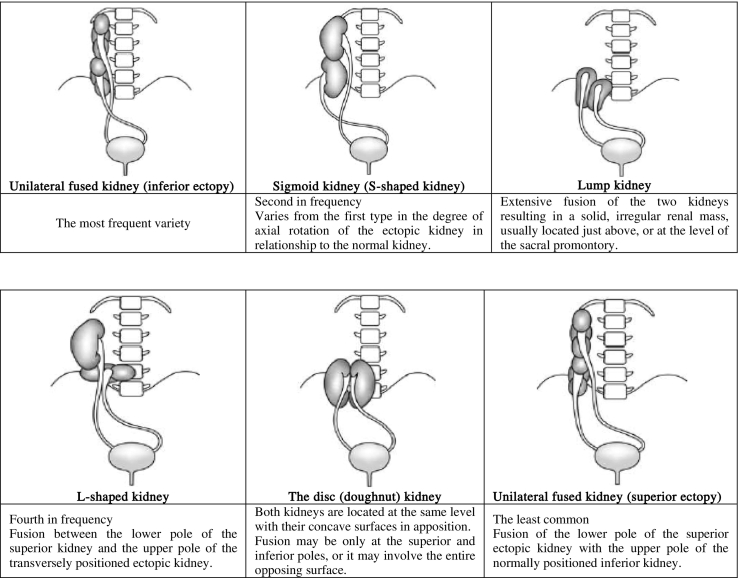
Classification of crossed fused ectopia.

Since the first case described in 1654 by Pamarolus [7], several theories have been suggested to elucidate the origin of CFRE. Many authors suggested the ureteral theory, in which there is aberrant migration and crossing of the midline by the metanephric blastema and the ureteral bud, mostly during the fourth to eighth week of gestation [8]. The mechanical theory proposes that during cranial ascent, the kidneys pass through the arterial fork between the two umbilical arteries. Hence, any abnormal position of umbilical arteries may squeeze nephrogenic blastemas close together allowing their fusion, and result in abnormal renal migration to the contralateral side following the path of least resistance [8].

CFRE remains asymptomatic throughout the patient's lifetime, and it is detected incidentally during autopsy and radiological or surgical interventions. Flank pain is the most common complaint [2], [4], due to possible dragging on the renal vessels by the weight of the renal unit [9]. Other symptoms can be reported such as dysuria, hematuria and recurrent urinary tract infections [10].

The most frequent physical finding is a palpable abdominal mass, mainly when the renal ectopy is associated with hydronephrosis [4], [10]. Hydronephrosis can be caused by vascular structures originating from the renal atypical pedicle, forming a vascular tie [11]. In our case, hydronephrosis with obstructive pyelonephritis in the orthotopic kidney was caused by the ectopic kidney itself which was hydronephrotic by an obstructive ureteral calculus. To our knowledge, this is the first case to be reported in the literature.

Imaging has a key role in diagnosing CFRE. Ultrasonography (USG) may show characteristic anterior and/or posterior notch with different orientation of the two collecting systems and absence of a kidney in the contralateral fossa [7]. It can also detect hydronephrosis, calculi and dysplasia. When combined with Doppler, vascular study of arterial supply and veinous drainage can be given. Contrast enhanced computed tomography (CT) has high sensitivity in making diagnosis, and accurate mapping of vascular supply [7].

CFRE may be associated with pathological conditions like renal calculi, hydronephrosis, vesico ureteral reflux and renal neoplasms [12]. Possible association with skeletal (spina bifida, scoliosis, radial cub hand) and gastrointestinal anomalies (imperforate anus and oesophageal atresia with tracheoesophageal fistula) has been also been described [13].

Vascular anatomy of the kidney is known to be atypical and may show rich variations [14]. A characteristic feature of CFRE is its vascular anatomy that is particularly uncertain and very variable. In fact, this feature is consistent with renal migration anomalies. During embryological ascent, the metanephros derives their blood supply from neighbouring vessels: Initially middle sacral artery, followed by common iliac and inferior mesenteric, and finally the abdominal aorta [6]. As the kidneys reach their final retroperitoneal position, former vessels degenerate to make place for permanent renal arteries and veins. If migration is arrested at any point, the temporary blood vessels become permanent, originating accessory renal arteries [12]. As a result, ectopic kidneys are often supplied by small anarchic vessels with both abdominal and pelvic origin, depending of the moment of the interruption of renal ascent. It is usually difficult to differentiate the main renal arteries, and the additional renal arteries. Total number of arteries can also vary from one to six [7], and they penetrate in both renal parenchyma renal sinus, and outside them [15].

CFRE, on and of itself, does not indicate a poor prognosis. The therapy of CFRE is usually aimed toward the complications of the condition rather than the treatment of the ectopic kidney itself. Complications of obstruction with calculus formation and infection, hematuria, and uremia may be expected with this aberrant condition, just as in other forms of fused kidney. In addition, it has been pointed out that aberrant kidney may show the following histological features: immature glomeruli, cystic changes (which may be haemorrhagic), and enlarged dilated tubules, or evidence of longstanding renal disease. In other cases, there may be evidence of infarction secondary to an abnormal blood supply [16]. All these histological varieties may be the factor promoting cyst formation, whose enlargement may ultimately bring about renal failure. Early diagnosis of potential complications that can accompany this anomaly is encouraged to prevent permanent renal damage [12]. Knowledge of such variations is crucial prior to performing any surgical or radiological intervention. Another challenging point in surgical management of CFRE, is that the parenchyma of both renal units are fused, thus, nephrectomy of aberrant kidney has to be performed through the parenchyma similar to a nephron sparing surgery [7].

## 4 Conclusion

Crossed fused renal ectopia is uncommon renal anomaly, mostly asymptomatic. The present case exposed that hydronephrosis of the ectopic kidney is frequent; however, it may lead to the obstruction of the orthotopic kidney, with pyelonephritis and sometimes severe complications. Heminephrectomy should be performed, with careful vascular approach. Preoperative radiological mapping of urinary tracts and renal vascularization may help to predict the difficulties that a surgeon may encounter.

## Ethical approval

N/a

## Sources of funding

This research did not receive any specific grant from funding agencies in the public, commercial, or not-for-profit sectors.

## CRediT authorship contribution statement

Amine Hermi: Data collection, Manuscript writing, Results discussion

Mokhtar Bibi: Manuscript writing and revision

Kheireddine Mrad Dali: Manuscript writing and revision

Houssem Hadj Alouane: Paper revision

Sami Ben Rhouma: Paper and figures revision

Yassine Nouira: Paper revision

## Guarantor

Amine Hermi is the guarantor of the study and accept full responsibility for the work and/or the conduct of the study, had access to the data and controlled the decision to publish.

## Consent

Written informed consent was obtained from the patient for publication of this case report and accompanying images. A copy of the written consent is available for review by the Editor-in-Chief of this journal on request.

## Declaration of competing interest

Authors do not report any conflict of interest.
